# S-Adenosylmethionine-Dependent Methylation, Protein Arginine Methyltransferases and Cardiovascular Diseases

**DOI:** 10.3390/biom16091365

**Published:** 2026-09-19

**Authors:** Zi Yan, Wenhui Zhao, Naixin Zhao, Jianguo Li

**Affiliations:** 1 Department of Physiology, Shanxi Medical University, Taiyuan 030001, China; yanzi1@sxmu.edu.cn (Z.Y.); dr20240041107@sxmu.edu.cn (W.Z.); zhaonx2025@mail.sustech.edu.cn (N.Z.); 2 MOE Key Laboratory of Cellular Physiology, Shanxi Medical University, Taiyuan 030001, China; 3 Shanxi Key Laboratory of Aging-Related Diseases Mechanisms and Translation, Taiyuan 030001, China

**Keywords:** S-adenosylmethionine (SAM)-dependent methyltransferases, protein arginine methyltransferases, cardiovascular diseases, post-translational modification

## Abstract

Methylation, catalyzed by a diverse family of S-adenosylmethionine (SAM)-dependent methyltransferases, is a fundamental biochemical process that regulates the function of proteins, DNA, and RNA. Among these modifications, protein arginine methylation, catalyzed by protein arginine methyltransferases (PRMTs), represents a critical post-translational mechanism regulating transcription, DNA repair, RNA splicing, and signal transduction. Emerging evidence highlights important roles of PRMTs in cardiovascular diseases (CVDs); however, current research is limited by several challenges, including the paradoxical and context-dependent functions of individual PRMTs and the lack of highly specific inhibitors or activators, leaving their therapeutic potential incompletely defined. This review summarizes the classification, structure, and functional characteristics of methyltransferases, with a particular focus on PRMTs and their roles in normal physiology and diverse CVDs. We integrate current knowledge to provide a comprehensive understanding of how PRMTs contribute to cardiovascular pathophysiology by methylating histones, transcription factors, and other functional proteins. Collectively, PRMTs emerge as pivotal regulators in CVD development and progression. Future studies should prioritize resolving the functional duality of specific PRMTs, developing targeted precision therapeutics, and elucidating the roles of less-characterized family members in CVDs.

## 1. Introduction

Methylation is a universal biochemical reaction in living organisms that is catalyzed by methyltransferases and plays important roles in many biological processes, including the regulation of the expression and functions of proteins, DNA, RNA, and other biomolecules [1,2]. S-adenosylmethionine (SAM, also known as AdoMet) serves as the main methyl donor in most methylation reactions and is utilized by SAM-dependent methyltransferases [3,4]. Protein methylation is a major form of posttranslational modification that influences protein stability, enzymatic activity, cellular localization, and molecular interactions. By altering protein hydrophobicity and steric properties, methylation modulates the functions of histones and numerous non-histone proteins involved in gene regulation and diverse cellular processes [5,6]. Among methylation modifications, arginine methylation, catalyzed by protein arginine methyltransferases (PRMTs), represents a predominant and functionally important modification [7,8]. Dysregulated PRMT-mediated arginine methylation has been implicated in the pathogenesis of multiple diseases, including cardiovascular diseases (CVDs), cancer, neurodegenerative diseases, inflammation, and antiviral responses [9,10,11,12]. In this review, we first systematically summarize the classification and biological functions of different methyltransferases. Based on this, we then refine our focus to the PRMTs family and dive into their emerging roles in CVDs.

## 2. Types of SAM-Dependent Methyltransferases

**Figure 1:**
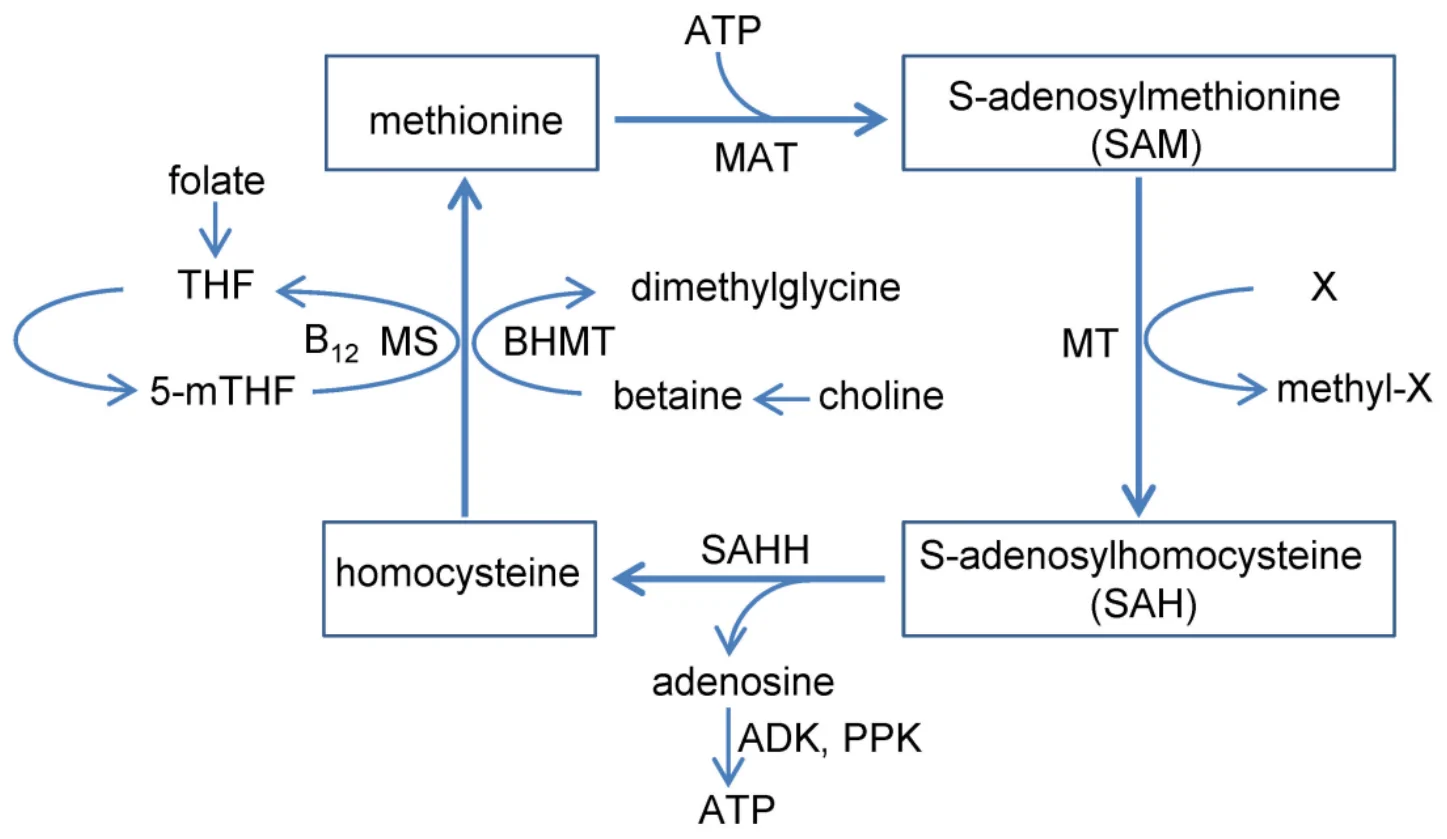
Methylation cycle. Folate, betaine, and choline come from food. MT, SAM-dependent methyltransferase; ADK, adenosine kinase; PPK, polyphosphate kinase; ATP, adenosine triphosphate; 5-mTHF, 5-methyltetrahydrofolate; THF, tetrahydrofolate.

Methyltransferases catalyze the transfer of a methyl group from SAM to the C, O, N, or S atoms of an acceptor molecule, generating S-adenosylhomocysteine (SAH or AdoHcy) as a byproduct [9]. SAH is a potent competitive inhibitor of many methyltransferases [13,14]. This methylation reaction (SAM + acceptor → SAH + methylated product) has a relatively low standard free energy change (ΔG°), making SAM a highly efficient methyl donor compared with other methyl sources, such as folate, methionine, betaine, or choline [13]. Through the coordinated activations of folate, vitamin B_12_ (B_12_), betaine, choline, adenosine triphosphate (ATP), and enzymes including SAH hydrolase (SAHH), betaine homocysteine S-methyltransferase (BHMT), methionine synthase (MS), and methionine adenosyltransferase (MAT), SAH can be efficiently recycled back to SAM via the intermediate homocysteine and methionine. Folate, betaine, and choline are largely obtained through dietary intake (Figure 1) [15,16]. Notably, this review excludes SAM-independent methyltransferases that do not utilize SAM as a methyl donor, such as bacterial L-carnitine methyltransferase (mtcB), which transfers methyl groups from l-carnitine to B_12_ or folate [17].

The methyltransferase superfamily can be classified according to either substrate specificity or the structural and biochemical features of methyltransferase [1,18].

### 2.1. Classification Based on Substrate Specificity

#### 2.1.1. Protein Methyltransferases

Protein methyltransferases are the most extensively studied methyltransferases and catalyze the methylation of protein substrates, including histones and non-histones [19,20,21]. Protein methylation mainly occurs on lysine and arginine residues and is catalyzed by protein lysine methyltransferases (PKMTs) and protein arginine methyltransferases (PRMTs), respectively [12,22]. Protein methylation also occurs at other amino acid residues, further expanding the diversity and regulatory complexity of protein methylation [7,8].

Given that the dysregulation of PRMTs is associated with various diseases, PRMTs have emerged as promising therapeutic targets. To date, multiple PRMT inhibitors have been investigated, and several candidates are undergoing clinical trials. More detailed information has been reviewed elsewhere [23,24,25].

#### 2.1.2. DNA Methyltransferases

DNA methyltransferases (DNMTs) catalyze DNA methylation, a major epigenetic modification involved in regulating gene expression, genomic imprinting, and chromatin organization [26]. DNA methylation predominantly occurs as 5-methylcytosine (m^5^C) at cytosine–phosphate–guanine (CpG) dinucleotide sites. The methylation of CpG sites within the promoter regions is generally associated with transcriptional silencing [26]. The DNMT family includes DNMT1, DNMT2, DNMT3A, DNMT3B, and DNMT3L. DNMT1 maintains the pre-existing methylation status during DNA replication. Although DNMT2 shares strong sequence similarity with other DNMT family members, it functions predominantly as an RNA methyltransferase. DNMT3A and DNMT3B mediate de novo methylation of previously unmethylated CpG sites, whereas DNMT3L lacks a catalytic domain but can bind to DNMT3A and DNMT3B and enhance their functions [1,27].

#### 2.1.3. RNA Methyltransferases

RNA methylation plays critical roles in regulating post-transcriptional and translational processes. RNA methyltransferases catalyze the methylation of diverse RNA species, including messenger RNA (mRNA), ribosomal RNA (rRNA), transfer RNA (tRNA), microRNA (miRNA), small nuclear RNA (snRNA), small nucleolar RNA (snoRNA), and long noncoding RNA (lncRNA). Common RNA methylation marks include 6-methyladenosine (m^6^A), 5-methycytosine (m^5^C), 1-methyladenosine (m^1^A), 3-methylcytosine (m^3^C), and 7-methylguanosine (m^7^G) [28,29]. These modifications are catalyzed by several RNA methyltransferase families, including methyltransferase-like (METTL) protein, tRNA methyltransferase (TRMT), the NOL1/NOP2/SUN domain (NSUN) family, and DNMT2 [28,29].

#### 2.1.4. Natural Product Methyltransferases

Natural-product methyltransferases (NPMTs) catalyze the methylation of small molecules and play essential roles in the biosynthesis of natural products by modifying their biological and physical properties [30]. Based on the atom receiving the methyl group, NPMTs are classified as O-methyltransferase (OMT), N-methyltransferase (NMT), C-methyltransferase (CMT), and S-methyltransferase (SMT) [30]. For example, catechol O-methyltransferase (COMT) degrades catechol neurotransmitters such as dopamine in the brain, whereas phenylethanolamine N-methyltransferase (PNMT) catalyzes the methylation of norepinephrine to form epinephrine, an important hormone involved in stress response [1].

### 2.2. Classification Based on the Structural and Biochemical Features of Methyltransferases

Methyltransferases can also be classified into five evolutionarily distinct classes (Classes I–V) based on their structural and biochemical features [18,30,31].

#### 2.2.1. Class I Methyltransferases

Class I methyltransferases contain a characteristic Rossmann fold motif consisting of a seven-stranded β-sheet flanked by α-helices on both sides. The central topological switch-point in this αβα sandwich forms a deep cleft for SAM binding. Class I enzymes constitute the largest methyltransferases group that catalyzes the methylation of proteins, DNA, RNA, and many small molecules [18,32].

#### 2.2.2. Class II Methyltransferases

Class II methyltransferases contain a long antiparallel β-sheet at the center of the protein, surrounded by helices that form a horseshoe-like topology. SAM binds within a shallow groove along the edge of the central β-sheets [18]. Methionine synthase (MS) is the only representative methyltransferase of this class. It utilizes the folate-derived methyl group to methylate homocysteine and generate methionine, requiring vitamin B_12_ as a cofactor [30].

#### 2.2.3. Class III Methyltransferases

Class III methyltransferases contain two αβα-domains, each composed of five β-strands and four α-helices, which together form a large SAM-binding pocket. In bacteria, uroporphyrinogen-III C-methyltransferase (CobA) and siroheme synthase (CysG) participate in the biosynthesis of vitamin B_12_ and siroheme through a methylation reaction [30,33]. Interestingly, recent studies have shown that gut microbes can affect gut–brain communication by producing vitamin B_12_, which reduces cholinergic signaling [34].

#### 2.2.4. Class IV Methyltransferases

Class IV methyltransferases possess a Class I-like αβα sandwich (Rossmann fold motif) but differ in the C-terminus, which folds back to form a knot-like structure for SAH binding [18]. This class of methyltransferases includes the SPOUT family, the second most abundant group of methyltransferases, many of which function as RNA methyltransferases [35].

#### 2.2.5. Class V Methyltransferases

Class V methyltransferases contain nine curved β strand, and a C-terminal knot-like structure resembling that of Class IV enzymes, creating a cavity for SAH binding [18]. The Su(var)3-9, enhancer of zeste, and trithorax (SET)-domain methyltransferases represent the most prominent members of this class [18,30]. SET domain-containing methyltransferases catalyze the methylation of lysine residues on histones and other proteins and are therefore classified as protein lysine methyltransferases (PKMTs) [36,37].

### 2.3. Radical SAM Methyltransferases

The conventional methyltransferases discussed in this review catalyze methylation through a bimolecular nucleophilic substitution reaction mechanism. In contrast, another class of methyltransferases, known as radical SAM methyltransferases, mediates methylation through radical-based chemical reaction, utilizing the 5′-deoxyadenosyl radical as a key catalytic intermediate [38]. Radical SAM methyltransferases belong to the broader radical SAM superfamily and comprise at least five distinct classes. A comprehensive overview of radical SAM methyltransferases and their catalytic mechanisms have recently been reviewed elsewhere [31,39].

Overall, methyltransferases catalyze the transfer of methyl groups from SAM to diverse substrates, including proteins, DNA, RNA, and small molecules, generating SAH as a byproduct that can subsequently be recycled to generate SAM. Based on substrate specificity, methyltransferases can be classified as protein, DNA, RNA, and natural-product methyltransferases. Based on their structural and biochemical characteristics, methyltransferases are further divided into five major classes (I–V).

Since SAM and SAH are dynamically balanced through the methionine cycle, drugs targeting methyltransferases might trigger feedback metabolic adaptations. Therapies targeting key enzymes in the methionine cycle (such as SAHH or MAT) or adjusting dietary nutrient intake could potentially enhance the effectiveness of methyltransferase interventions. In addition, specific drugs could be developed targeting the unique structural characteristics of different types of methyltransferases, such as the Rossmann fold domain, knot-like structure, or SET domain. Finally, investigating methylomics as an information hub that mediates gene–environment interactions, with its scope encompassing the methylation patterns of DNA, RNA, protein methylation, and small-molecule factors, will provide an important basis for understanding development, aging, and disease.

## 3. Protein Arginine Methyltransferases

Methylation normally occurs at sulfur (S) atoms of methionine and cysteine, oxygen (O) atoms of aspartate and glutamate, and nitrogen (N) atoms of asparagine, histidine, proline, alanine, lysine, and arginine residues in both histones and non-histone proteins [7]. However, protein methylation occurs predominantly on lysine and arginine residues and is catalyzed by PKMTs and PRMTs, respectively [7,8].

**Figure 2:**
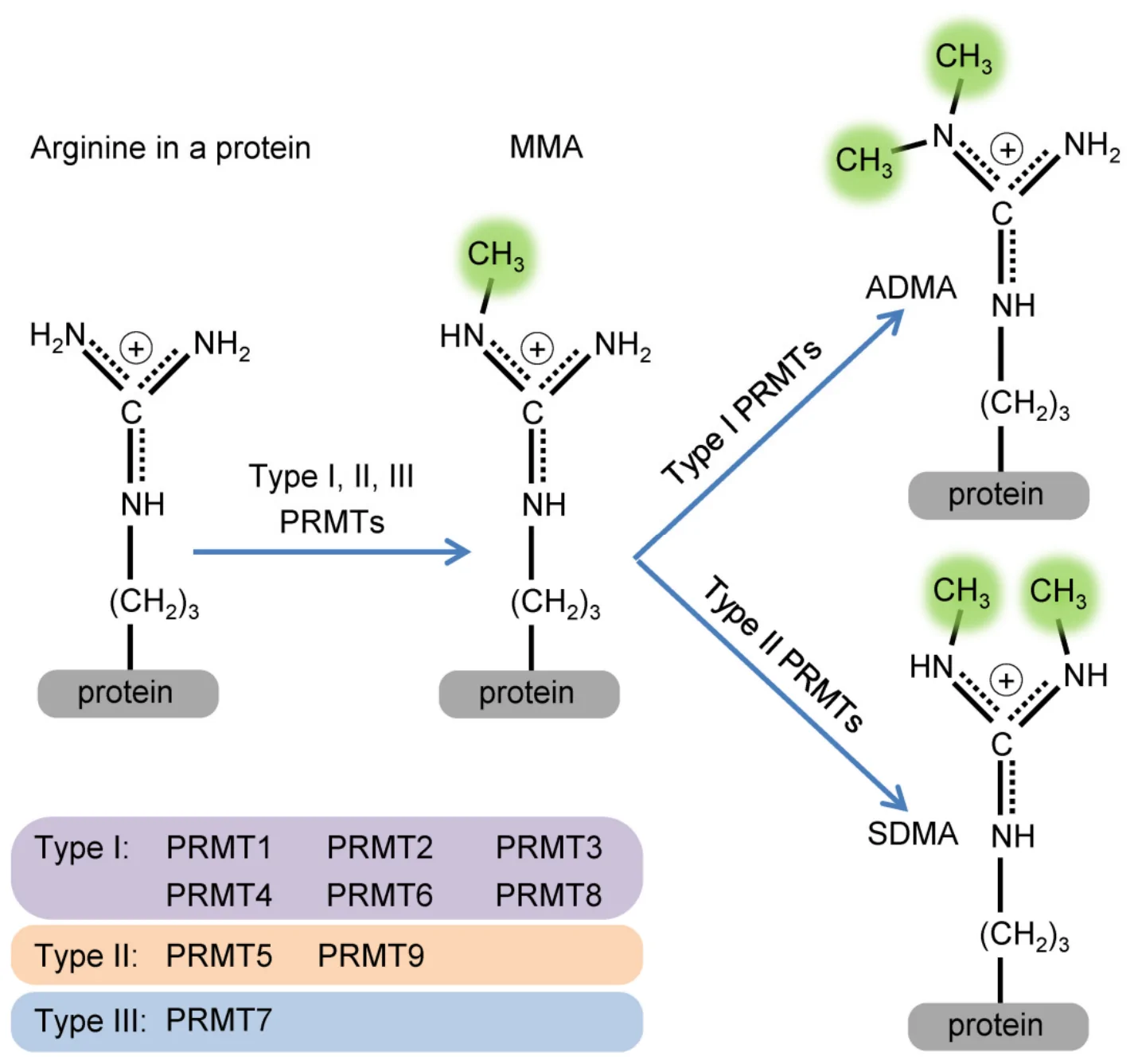
Schematic of the methylated nitrogen of arginine residues in protein according to three types of PRMTs. Type I PRMTs catalyze the formation of MMA and ADMA. Type II PRMTs catalyze the formation of MMA and SDMA. Type III PRMT catalyzes the formation of MMA.

The human genome encodes approximately 100 putative PKMTs, and their structures and functions have been extensively reviewed elsewhere [40,41,42]. In this section, we focus on the structures and physiological functions of PRMTs, which contain nine members in humans [12]. Based on the methylarginine species generated during catalysis, PRMTs are classified into three types. Type I PRMTs, including PRMT1, 2, 3, 4, 6, and 8, catalyze the formation of monomethylarginine (MMA) and asymmetric dimethylarginine (ADMA). Type II PRMTs, including PRMTs 5 and PRMT9, catalyze the formation of MMA and symmetric dimethylarginine (SDMA). PRMT7 is the only Type III PRMT that catalyzes the formation of MMA (Figure 2) [12,43].

### 3.1. The Structures of PRMTs

**Figure 3:**
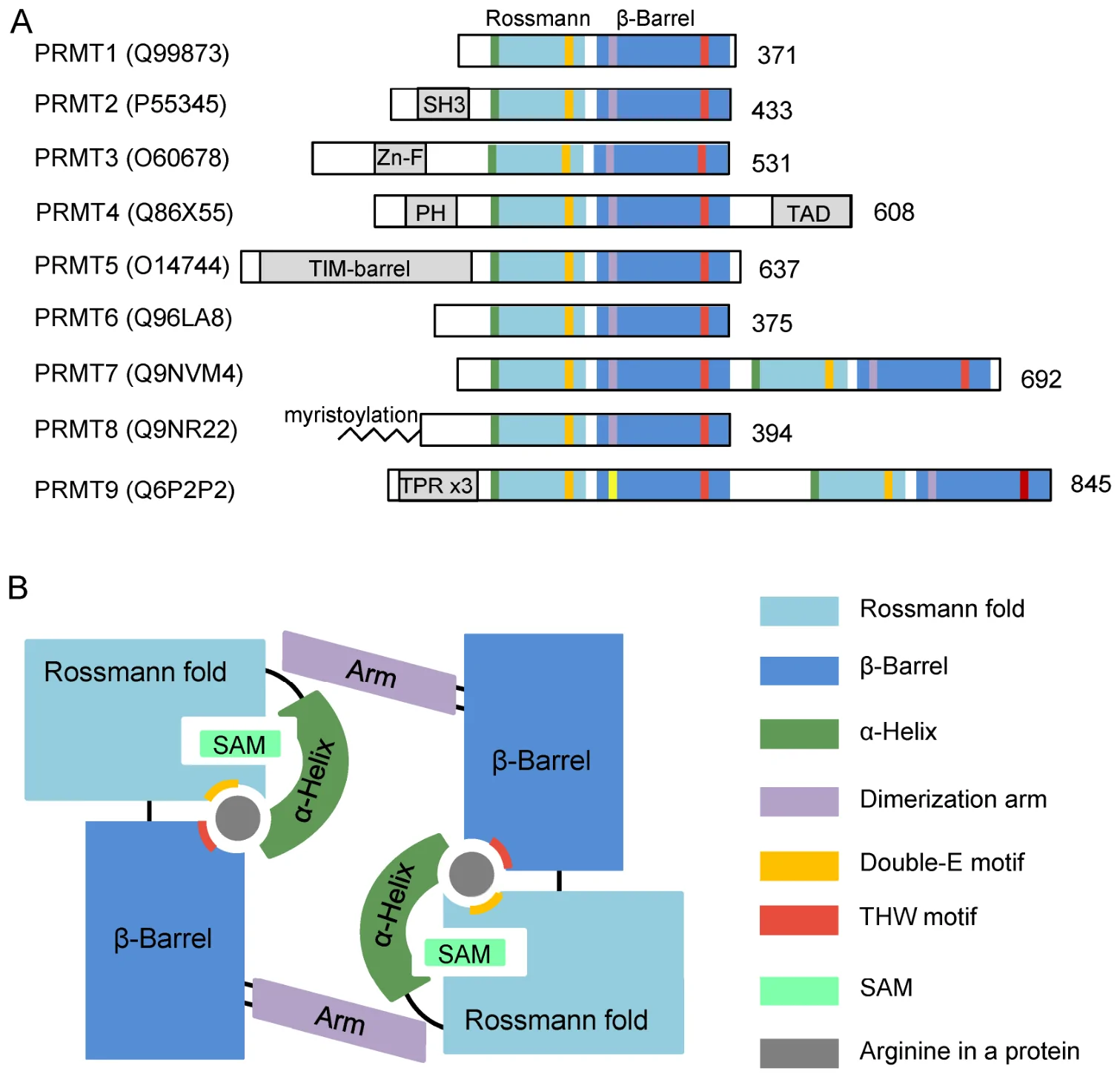
Structural overview of PRMTs. (**A**) Domain arrangement of human PRMTs. Nine PRMTs are identified. UniProt accession numbers are shown in parentheses for all PRMTs. (**B**) Schematic diagram of a typical PRMT dimer structure mechanism. The Rossmann fold and its associated α-helix form the SAM-binding pocket. The Rossmann fold, α-helix, and β-barrel together create the arginine substrate-binding pocket. The double-E motif within the Rossmann fold and the THW motif within the β-barrel are important for substrate binding. The dimerization arm projecting from one β-barrel interacts with the Rossmann fold of the opposing subunit.

Human PRMTs share two highly conserved structural modules: a Rossmann fold domain, which contains α-helical elements and mediates SAM binding, and a β-barrel domain containing the dimerization arm [44,45,46,47,48]. Although PRMTs possess conserved structural cores, substrate specificity is largely determined by their distinct N-terminal domains. The differences in the N-terminal include a SRC homology 3 (SH3) domain in PRMT2, a zinc-finger in PRMT3, a pleckstrin homology (PH) domain in PRMT4 (also known as coactivator-associated arginine methyltransferase 1, CARM1), a triose-phosphate isomerase (TIM)-barrel in PRMT5, an N-myristoylation domain in PRMT8, and three tetratricopeptide repeat (TPR) motifs in PRMT9 (Figure 3A) [25,47]. At the C-terminal, PRMT4 contains a transcriptional activation domain (TAD) that enhances the transcriptional activation of nuclear receptors, whereas PRMT7 and PRMT9 each contain a second PRMT domain that contributes to pseudodimer formation [25,47].

Type I and type II PRMTs generally require homodimerization for full catalytic activity. The Rossmann fold and its associated α-helix form the SAM-binding pocket, whereas the Rossmann fold, α-helix, and β-barrel together create the arginine substrate-binding pocket. Conserved motifs, including the double-E motif within the Rossmann fold and the THW motif within the β-barrel, are important for substrate binding and catalysis. In addition, the dimerization arm projecting from one β-barrel interacts with the Rossmann fold of the opposing subunit, stabilizing the active dimeric configuration (Figure 3B) [45,46,47,48]. In contrast, type II PRMT9 and type III PRMT7 contain tandem PRMT domains and form intramolecular pseudodimers. In these enzymes, the C-terminal domain of the pseudodimer is catalytically inactive but remains essential for overall enzymatic activity [46,49]. PRMT5 typically exists as a hetero-octamer with MEP50/WDR77, whereas PRMT7 and PRMT9 function primarily as monomers [9,50,51]. Furthermore, certain PRMTs are capable of forming heterodimeric or higher-order oligomeric complexes, further expanding the structural and functional diversity of this enzyme family [47,52].

### 3.2. Overall Physiological Functions of PRMTs

PRMTs regulate many fundamental cellular processes by methylating arginine residues on histone or non-histone proteins, including gene transcription, RNA processing, signal transduction, and the DNA damage response [53,54]. Arginine plays critical roles in nucleic acid binding and protein–protein interactions through its guanidinium group. This guanidinium group consists of a central carbon atom bonded to three nitrogen atoms, forming a highly stable protonated guanidinium cation under physiological conditions. This unique chemical structure enables arginine to form hydrogen bonds and π interactions with nucleic acids and proteins [53,55,56]. PRMT-mediated methylation modifies the hydrogen-bonding capacity and steric properties of arginine side chains through the addition of one or two methyl groups, thereby altering the interaction of methylated arginine residues with proteins and nucleic acids [53,57].

Due to their deep substrate-binding pocket, PRMTs preferentially methylate arginine residues located within the intrinsically disordered regions (IDRs), which are structurally flexible segments often found in the N- and C-terminal regions of proteins [58]. The arginine-containing motifs targeted by PRMTs exhibit distinct sequence preferences. Most PRMTs preferentially methylate glycine-arginine-rich (GAR) motifs, whereas PRMT4 prefers proline-glycine-methionine (PGM) motifs and PRMT7 methylates arginine residues within the RXR motifs, in which two arginine residues are separated by a single amino acid [12,59,60]. Proteins containing methylated arginine residues within IDRs generally play important roles in both nucleic-acid-binding and protein–protein interactions, and are increasingly recognized as key regulators of biomolecular condensates [61].

#### 3.2.1. Type I PRMTs

PRMT1 is the predominant protein arginine methyltransferase in mammalian cells and is responsible for the majority of cellular arginine methylation, accounting for more than 85% of total arginine methylation [24]. PRMT1 regulates numerous physiological processes, including transcription, DNA repair, RNA splicing, and signal transduction [12,62]. Alternative splicing generates at least seven PRMT1 isoforms, which differ in their enzymatic activity, subcellular localization, and substrate specificity [63].

PRMT2 was originally identified as a coactivator of estrogen and androgen receptors [64,65]. However, subsequent studies have demonstrated that PRMT2 can also function as a transcriptional repressor depending on the cellular context. The SH3 domain mediates protein–protein interactions and contributes to the biological functions of PRMT2 [66,67,68].

PRMT3 regulates ribosome biogenesis and homeostasis through the methylation of ribosomal protein S2 (RPS2), which is recognized by its N-terminal zinc-finger domain [69]. This domain is critical for substrate recognition and intracellular localization, enabling PRMT3 to participate in diverse cellular processes, including gene expression and metabolic regulation [70,71].

PRMT4, also known as CARM1, functions as a transcription coactivator and regulates diverse biological processes, including cellular differentiation, oxidative stress responses, and cell death [72]. The pleckstrin homology (PH) domain contributes to substrate recognition and enzymatic activity, and its deletion significantly impairs PRMT4 functions [59]. In addition, CARM1 plays important roles in cardiomyocyte maturation and cardiac development [73].

PRMT6 is primarily localized in the cell nucleus, unlike PRMT3 and PRMT5, which are mainly found in the cytoplasm [74]. PRMT6 regulates gene expression through histone methylation, acting either as a transcriptional repressor via histone H3 arginine 2 asymmetric dimethylation (H3R2me2a) or as a transcriptional activator through the asymmetric dimethylation of histone H3 arginine 42 (H3R42me2a) at gene promoters. PRMT6 also methylates many non-histone proteins involved in diverse cellular pathways [74,75].

PRMT8 is primarily expressed in the central nervous system and is uniquely associated with the plasma membrane through an N-terminal myristoylation motif [76]. Although structurally similar to PRMT1, PRMT8 exhibits distinct substrate preferences and physiological functions, particularly in neuronal development, synaptic plasticity, neurotransmission, and neuronal stress adaptation [76,77].

#### 3.2.2. Type II PRMTs

PRMT5 contains an N-terminal TIM-barrel domain that mediates interaction with methylosome protein 50 (MEP50, also known as WDR77) [78]. MEP50 is an essential cofactor that enhances substrate recognition and catalytic activity of the PRMT5 complex [46,79]. PRMT5 methylates Smith antigen (Sm) proteins, promoting their assembly into mature small nuclear ribonucleoproteins (snRNPs) which are required for pre-mRNA splicing [80]. Structural studies have demonstrated that phenylalanine 327 (F327) within the catalytic pocket is critical for the generation of SDMA [78].

PRMT9 contains two tandem PRMT domains and three N-terminal TPR motifs which mediate protein–protein interactions [11]. PRMT9 methylates splicing factor 3b subunit 2 (SF3B2) and regulates alternative splicing through its effects on snRNPs maturation and the interaction with the pre-mRNA anchoring site [51,81].

#### 3.2.3. Type III PRMT

PRMT7 is the sole member of the type III PRMT and exclusively catalyzes the formation of MMA [82]. PRMT7 contains two tandem PRMT domains that cooperate to maintain its catalytic activity, although only one domain is catalytically active [82]. PRMT7 participates in transcriptional regulation, snRNP maturation, and alternative splicing. Its unique ability to generate only MMA results from a restricted active-site architecture that prevents the formation of dimethylarginine products. Notably, the mutation of residues within the conserved double E-loop can switch PRMT7 to a type I or type II PRMT [83]. In addition, PRMT7 activates PRMT5-mediated methylation of H4R3 through the prior methylation of histone H4 at R17, highlighting functional crosstalk between PRMT family members [84].

PRMT-mediated arginine methylation marks are read by Tudor domains with an aromatic-binding cage structure. Survival motor neuron (SMN) and Tudor domain-containing 3 (TDRD3) are the best studied Tudor domain-containing proteins. The Tudor domain of SMN binds SDMA over ADMA. The Tudor domain of TDRD3 recognizes ADMA over SDMA and MMA. SMN binds the spliceosomal core Sm proteins and acts as chaperone to regulate pre-mRNA splicing. TDRD3 forms a complex with topoisomerase IIIB (TOP3B), which is recruited to promoters to resolve the R-loops that form after RNA polymerase II [58].

In this section, we discussed the structural features and physiological functions of PRMTs. The nine members of PRMT are classified into three groups (Types I–III) based on the methylarginine species they generate. Despite sharing highly conserved catalytic domains, PRMTs achieve substrate specificity through distinct structural elements, particularly within their N-terminal regions. Through the methylation of arginine residues on IDRs, PRMTs regulate a broad spectrum of cellular processes, including transcription, DNA repair, RNA splicing, and signal transduction. The dysregulation of PRMT activity has been implicated in numerous human diseases, highlighting their importance as both fundamental regulators of cellular physiology and promising therapeutic targets.

The diversity of the PRMT family means that it provides abundant targets for drug development and precise interventions. Although conserved SAM-binding pockets and catalytic domains among PRMTs remain conserved, divergent N-terminal domains enable subtype-selective inhibitor design. Compounds may target specific disease-driving PRMT isoforms (such as PRMT1) to suppress pathological activity while retaining essential physiological functions. Targeting protein–protein interaction interfaces (such as the MEP50-PRMT5 complex) may indirectly interfere with PRMT function. Furthermore, the expression levels of the different PRMTs and the methylation status (such as ADMA, SDMA, MMA) of specific substrates could serve as clinical biomarkers to guide precision treatment.

## 4. Roles of PRMTs in Cardiovascular Diseases

PRMTs have been implicated in many diseases, including CVDs, cancer, and neurodegenerative diseases [9]. In addition, the methylated arginine residues on proteins can be hydrolyzed to generate free forms of MMA, ADMA, and SDMA, which participate in CVDs and other diseases by inhibiting the production of nitric oxide (NO) from L-arginine [85]. Further information has been extensively reviewed elsewhere [86,87,88]. In this section, we focus on the potential roles of PRMTs in CVDs through the methylation of proteins.

CVDs have remained the leading cause of death worldwide and result in significant social healthcare costs [89,90]. The global top 10 cardiovascular causes of death are ischemic heart disease (IHD), ischemic stroke, intracerebral hemorrhage, hypertensive heart disease (HHD), rheumatic heart disease, arrhythmias (atrial fibrillation and flutter), subarachnoid hemorrhage, other cardiomyopathy (such as hypertrophic cardiomyopathy and dilated cardiomyopathy), other cardiovascular diseases, and aortic aneurysm [89].

### 4.1. Ischemic Heart Disease

About half of cardiovascular deaths are caused by IHD [89]. The pathophysiological paradigm of IHD includes coronary atherosclerosis, inflammation, endothelial cell (EC) and vascular smooth muscle cell (VSMC) dysfunction, ischemic cardiomyocyte injury or death, and cardiac fibroblast proliferation [91,92,93].

#### 4.1.1. Atherosclerosis

Excess cholesterol leads to the formation of oxysterol, which stimulates the expression of ATP-binding cassette transporter A1 (ABCA1), thereby promoting the excretion of cholesterol and other lipids from the macrophages [94]. Liver X receptors (LXRs) are a class of nuclear receptors and transcription factors activated by oxysterol, which regulate gene expression related to cholesterol metabolism, fatty acid metabolism, and inflammation [95,96]. The overexpression of PRMT2 inhibits the formation of foam cells induced by ox-LDL in macrophages through the increased LXR ligand-dependent expression of ABCA1 which mediates cholesterol efflux [97,98]. This PRMT2-mediated LXR/ABCA1 axis is believed to be an important approach to preventing atherosclerosis.

It has been reported that the expressions of PRMT1 and PRMT3 were upregulated in the myocardial tissue of patients with atherosclerosis [99]. PRMT3 increased the activity of LXR by directly binding to it in a methylation-independent manner. In addition, overexpression of PRMT3 enhanced the expression of lipogenic proteins in the liver [100]. Studies have linked variation in the PRMT3 gene to changes in carotid intima-media thickness (CIMT), which is an indicator of subclinical atherosclerosis [101]. However, a recent study revealed that inhibiting PRMT3 reduced hepatic steatosis through LXR, without the negative impact on atherosclerosis susceptibility in hyperlipidemic mice [102]. This suggests that PRMT3 has a tissue-specific effect on LXR signaling. In macrophages, PRMT3 modulates LXR-mediated anti-atherosclerotic actions; in the liver, however, they promote triglyceride synthesis.

Sterol regulatory element-binding proteins (SREBPs) are transcription factors that regulate the expression of genes involved in lipid synthesis [103]. Among them, SREBP1a mediates lipid synthesis and growth. SREBP1c is associated with fatty acid synthesis and energy storage, and SREBP2 plays a role in cholesterol regulation [103]. PRMT5 promoted the activation of SREBP1a via methylation at R321, thereby increasing the lipid biosynthesis activity [104].

The expression of PRMT5 was increased in ox-LDL-stimulated human umbilical vein endothelial cell (HUVEC). Knockdown of PRMT5 alleviated apoptosis, inflammation, and endothelial dysfunction in ox-LDL-stimulated HUVECs by interacting with and suppressing the expression of programmed cell death 4 (PDCD4), which is involved in atherosclerosis progression [105]. Additionally, studies indicated that PRMT5 can inhibit the tumor suppressor function of PDCD4 through methylation at R110 in MCF7 cells [106]. Notably, PDCD4 deficiency enhanced the anti-tumor effect and anti-atherosclerosis effect of macrophages [107,108]. However, it is important to point out that direct biochemical evidence supporting PRMT5-dependent PDCD4 R110 methylation in atherosclerosis is currently absent.

#### 4.1.2. Inflammation

The C-X-C motif chemokine ligand 10 (CXCL10) is an important chemokine that contributes to inflammation, atherosclerosis, and coronary artery disease. PRMT5 increased the expression of CXCL10 through the methylation of the nuclear factor NF-Kappa-B p65 subunit (NF-κB p65) at both R30 and R35 [109]. The expression of another atherosclerosis-related chemokine, CXCL11, also required PRMT5 to methylate NF-κB p65 at R174 in EC [110]. PRMT5 also played a role in the inflammatory responses by increasing the expression of vascular cell adhesion molecule-1 (VCAM-1), which depended on either the methylation of NF-κB p65 at R30 in VSMC or the methylation of homeobox A9 (HOXA9) at R140 in EC [111,112]. These studies indicate that post-translational modifications of NF-κB p65 by PRMTs may serve as a primary driver for context-dependent gene transcription, and that PRMTs can modulate inflammation by modifying distinct transcription factors.

During myocardial injury, macrophages play important roles in the inflammatory response through distinct subtypes. They are divided into two groups: the M1 phenotype, which releases inflammatory factors, and the M2 phenotype, which exhibits anti-inflammatory activity and mediates tissue repair [113]. PRMT1 can induce aberrant MHC II transactivation in macrophages by methylating the Class II transactivator (CIITA), which in turn promoted inflammation and contributed to atherogenesis [114]. PRMT1 also promoted M2 macrophage polarization through the methylation of histones and non-histone proteins, which exhibited anti-inflammatory activity in atherosclerosis [115,116]. PRMT9 suppressed inflammation and ameliorated acute myocardial infarction by inhibiting the M1 macrophage polarization. This effect was achieved via PRMT9-mediated methylation of the signal transducer and activator of transcription 1 (STAT1) at R588 and R736, which further triggered STAT1 degradation [117].

#### 4.1.3. EC Dysfunction

EC is crucial for maintaining vascular homeostasis and usually underlies the development of atherosclerosis. Atheroprone oscillatory shear stress at arterial branches and curvatures is prone to atherosclerosis by increasing endothelial permeability [118]. Adhesion protein Kindlin-2 is a key regulator of integrin activation, cell migration, and adhesion with the extracellular matrix. PRMT5 inhibited Kindlin-2 through methylation at R290, thereby impairing EC barrier function. The Inhibition of PRMT5 attenuated the atheroprone oscillatory shear stress-induced EC barrier disruption and ameliorated atherosclerosis [119].

As a scaffold protein, PRMT5 promotes vascular morphogenesis by increasing the expression of angiogenesis-related genes in EC [120]. The inhibition of PRMT5 reduced the protein stability of hypoxia-inducible factor 1-α (HIF-1α) and the production of proangiogenic vascular endothelial growth factor (VEGF) in post-ischemic EC, thereby weakening angiogenesis [121]. PRMT5 inhibited apoptosis signal-regulating kinase 1 (ASK1) activity by methylating at R89, thus suppressing EC apoptosis [122]. PRMT4 promoted filopodia protrusions in endothelial cells by the methylation of VEGF receptor-2 (VEGFR-2) at R847 [123]. Another study showed that the overexpression of PRMT7 in EC enhanced revascularization by suppressing endoplasmic reticulum (ER) stress after myocardial ischemia [124].

#### 4.1.4. VSMC Dysfunction

The phenotypic switching of VSMC from a contractile state to a dedifferentiated synthetic state is involved in the development of atherosclerosis [93]. PRMT5 expression was increased in human atherosclerotic lesions tissues. The overexpression of PRMT5 promoted a synthetic state of VSMC for proliferation and migration, either through dimethylation of histone H3 arginine 8 (H3R8) and histone H4 arginine 3 (H4R3), or by stabilizing Kruppel-like factor 4 (KLF4) through methylation at R377 [125,126]. Methylated KLF4 inhibited the function of the serum response factor (SRF), which recruited myocardin to maintain the contractile state of VSMC [126].

#### 4.1.5. Ischemic Cardiomyocytes Injury or Death

The expression of PRMT4 was markedly increased in ischemic myocardial tissues and hypoxic cardiomyocytes [9,127]. The overexpression of PRMT4 promoted hypoxia-induced cardiomyocyte apoptosis by increasing caspase-3 expression [127]. PRMT7 expression was also increased in ischemic myocardial tissues from patients or mice. In addition, PRMT7 overexpression reduced apoptotic cardiomyocytes by suppressing ER stress after myocardial ischemia [124]. Studies have shown that PRMT7 can methylate p38 mitogen activated protein kinase (p38 MAPK) and heat shock protein 70 (HSP70), both of which are involved in ER stress [124,128]. ER stress-induced cardiomyocyte death was also inhibited by PRMT1 through the methylation of activating transcription factor 4 (ATF4) [129].

#### 4.1.6. Cardiac Fibroblast Proliferation

The downregulation of PRMT1 inhibited cardiac fibroblast proliferation in ischemic mice by reducing the production of reactive oxygen species (ROS) and neutrophil extracellular traps (NETs) through methylating Ras-associated protein-1 (RAP1) [130]. PRMT4 expression was markedly upregulated in ischemic heart tissue, and PRMT4 overexpression exacerbated cardiac fibrosis within the injured region [127].

### 4.2. Hypertensive Heart Disease

Apart from ischemic heart disease, ischemic stroke and intracerebral hemorrhage, HHD is the fourth leading cause of cardiovascular deaths [89]. HHD is defined as heart failure that is caused by the direct and long-term effects of unmanaged hypertension. Under pressure overload, cardiac hypertrophy is an important adaptive mechanism for the heart to maintain contractile strength and pumping function [131]. However, hypertension and valvular heart disease primarily lead to pathological cardiac hypertrophy and fibrosis, eventually resulting in heart failure [132,133].

#### 4.2.1. Cardiac Hypertrophy

Ca^2+^/calmodulin-dependent kinase II (CaMKII) plays important roles in cardiac function [134]. The knockdown of PRMT1 induced cardiomyocyte hypertrophy and increased CaMKII activity. The overexpression of PRMT1 inhibited the activity of CaMKII through the methylation of R9 and R275, thereby attenuating phenylephrine (PE)-induced cardiomyocyte hypertrophy [135]. The overexpression of PRMT1 also ameliorated isoproterenol (ISO)-induced cardiac hypertrophy by decreasing the expression of the CaMKIIδC isoform through methylating serine/arginine-rich splicing factor 1 (SRSF1) [136]. It has been reported that, in the abdominal aortic constriction model, cardiac hypertrophy was associated with reduced methylation of cardiac troponin I (cTnI) at R146/R148, which was catalyzed by PRMT1 [137]. CTnI is the inhibitory component of the troponin complex and its mutations are involved in hypertrophic cardiomyopathy (HCM) [138]. Additionally, the expression of PRMT1 was reduced in hypertrophic cardiac tissue from patients with acquired aortic valve stenosis [139].

The expression of PRMT5 was decreased in hypertrophic cardiomyocytes induced by ISO or transverse aortic constriction (TAC) [140,141,142]. The knockdown of PRMT5 exacerbated cardiomyocyte hypertrophy, whereas overexpression of PRMT5 alleviated ISO-induced cardiac hypertrophy by methylating HOXA9 and impairing its binding to the promoters of hypertrophy-related genes, including brain natriuretic peptide (BNP) and β-myosin heavy chain (β-MyHC) [140,143]. Another study found that overexpression of PRMT5 attenuated PE-induced cardiomyocyte hypertrophy by methylating GATA4 at R317 and blocking p300-mediated GATA4 acetylation [143]. The overexpression of PRMT5 also suppressed ISO-induced cardiomyocyte hypertrophy by increasing H4R3 symmetric methylation (H4R3me2s). Additionally, PRMT5-induced H4R3me2s ameliorated cardiomyocyte hypertrophy through the upregulation of filamin A interacting protein 1 like (FILIP1L) and subsequent enhancement of β-Catenin degradation [141]. Previous studies indicated that the overexpression of β-Catenin led to the progression of cardiac hypertrophy [144]. In addition, the overexpression of PRMT5 dramatically reduced angiotensin II (Ang II)-induced cardiac hypertrophy and fibrosis by impairing the E2F transcription factor 1 (E2F-1)/NF-κB/NLR family pyrin domain containing 3 (NLRP3) pathway [142]. Inhibition of NLRP3 alleviated TAC-induced cardiac hypertrophy and pyroptosis [145].

On the other hand, Katanasaka found that inhibition of PRMT5 alleviated TAC-induced cardiac hypertrophy [146]. In this study, cardiac hypertrophy was suppressed by PRMT5 deficiency in periostin-expressing fibroblasts [146]. Furthermore, fibroblasts produced a variety of paracrine factors that exerted pro-hypertrophic and anti-hypertrophic effects and acted on cardiomyocytes [147]. Therefore, these opposing effects of PRMT5 on cardiac hypertrophy might be related to its differential expression on different cell types.

PRMT6 expression was upregulated in PE and TAC-induced cardiomyocyte hypertrophy, and the overexpression of PRMT6 led to cardiomyocyte hypertrophy. Knockdown of PRMT6 inhibited PE-induced cardiomyocyte hypertrophy by reducing H3R2 asymmetric dimethylation (H3R2me2a), a key marker that modulates gene expression [148,149].

The knockout of PRMT7 induced cardiac hypertrophy in mice. The overexpression of PRMT7 suppressed the activation of β-Catenin proteins through methylation at residue R97, thereby attenuating Ang II-induced cardiomyocyte hypertrophy [150].

#### 4.2.2. Cardiac Fibrosis

PRMT1 promoted the activation of myofibroblasts by methylating nucleolar protein fibrillarin 1 (FBL), thus exacerbating cardiac fibrosis [151]. The overexpression of PRMT1 in cardiomyocyte inhibited CaMKII activity through methylation at R9 and 275, and then protected against cardiac fibrosis [135].

It has been reported that the differentiation of cardiac fibroblasts into myofibroblasts plays a critical role in cardiac fibrosis. The transforming growth factor-β (TGF-β)/Smad signaling pathway has been known to be involved in this process [152]. The depletion of PRMT5 in cardiac fibroblasts inhibited cardiac fibrosis and the expression of fibrosis-related genes in the TAC-treated mice, and PRMT5-mediated H3R2 dimethylation was required for fibrotic gene transcription induced by the TGF-β/Smad3 pathway [146]. On the other hand, another study showed that the overexpression of PRMT5 in cardiomyocytes reduced Ang II-induced cardiac fibrosis by impairing the E2F-1/NF-κB/NLRP3 pathway [142]. These studies indicated that PRMT1 and PRMT5 exert cell-type-dependent effects on cardiac fibrosis: their overexpression in cardiac fibroblasts promotes fibrotic responses, while the overexpression of these two enzymes in cardiomyocytes suppresses cardiac fibrosis.

Besides cardiac hypertrophy, the knockout of PRMT7 induced cardiac fibrosis in mice by activating β-Catenin [150]. Previous studies have indicated that wingless int1 (WNT)/β-Catenin signaling and TGF-β signaling pathways are two key interrelated pathways in regulating myofibroblasts [153].

### 4.3. Other CVDs

Beyond IHD and HHD, PRMTs are also implicated in other CVDs, including cardiomyopathies, arrhythmias, and aortic aneurysm (AA). Under most of these conditions, PRMTs exert protective effects. Notably, PRMT4 acts as a pathogenic exception, promoting cardiomyocyte ferroptosis in doxorubicin-induced cardiomyopathy (DIC) [154]. The detailed roles of distinct PRMTs in these diseases are discussed further below.

In patients with HCM, the expression of PRMT1 was reduced in the cardiac tissue, and PRMT1 prevents the mitochondria from undergoing Ca^2+^ overload through methylation-mediated suppression of mitochondrial Ca^2+^ uptake 1 (MICU1) [139].

Dilated cardiomyopathy (DCM) could be induced by the deletion of PRMT1 in mice, which exhibited aberrant cardiac alternative splicing, including the increased expression of the truncated isoform of eukaryotic translation initiation factor 4A2 (EIF4A2), which plays a role in stress granule formation. In addition, the truncated isoform of EIF4A2 was highly ubiquitinated in DCM mice [155]. The O-linked attachment of the N-Acetyl-beta-glucosamine (O-GlcNAc) modification of proteins regulates the function of the cardiovascular system [156]. The expression of PRMT5 was significantly decreased in the DCM patients. The cardiomyocyte-specific deletion of PRMT5 led to DCM in mice through promoting protein O-GlcNAcylation by increasing the aberrant alternative splicing isoform of N-acetyl-beta-glucosaminidase (OGA), which is responsible for removing O-GlcNAc from the protein [157].

Arrhythmogenic cardiomyopathy (ACM) can be caused by a genetic mutation in desmoplakin (R2834H). PRMT1 promotes the role of desmoplakin in intercellular junctions and cytoskeletal organization, which may happen through methylation at R2834 [158,159].

In the DIC model, PRMT4 inhibited glutathione peroxidase 4 (GPX4) by methylating nuclear factor erythroid 2-related factor 2 (NRF2), thereby enhancing ferroptosis [154]. PRMT5 was upregulated in doxorubicin -induced cardiac fibrosis. Furthermore, the overexpression of PRMT5 promoted TGF-β-induced Smad3 phosphorylation to activate cardiac fibroblast via methylation modification [160]. A recent study has shown that mice with cardiac-specific *Prmt7* ablation exhibited sex-specific cardiomyopathy. In addition, PRMT7 alleviated DIC by inhibiting JAK/STAT3 signaling pathway-mediated inflammation by increasing the suppressor of cytokine signaling (SOCS3) [161].

Arrhythmias are associated with the slow delayed rectifier K^+^ channel (I_Ks_) and the sodium voltage-gated channel alpha subunit 5 (Nav1.5) [162,163]. PRMT1 enhanced channel-phosphatidylinositol 4,5-bisphosphate (PIP_2_) interaction by methylating the potassium voltage-gated channel subfamily Q member 1 (KCNQ1) subunit of the I_Ks_, thereby upregulating the activity of I_Ks_ [163]. PRMT3 and PRMT5 increased the expression of Nav1.5 on the cell surface through methylating channels [164]. The previous study revealed that Nav1.5 was methylated at R526 or R680, which were mutation sites responsible for Brugada and long QT type 3 syndromes, respectively [165].

In patients with AA, the expression of PRMT1 and PRMT5 were downregulated, while the expression of PRMT7 was increased. Depletion of PRMT1 in VSMC led to VSMC apoptosis, elastic fiber fragmentation and collagen accumulation by attenuating the enrichment of H4R3me2a at the myocardin promoter region, thus causing aortic dilation and aortic dissection [166].

In this section, the link between PRMT1-PRMT7 and CVDs is described (Table 1). The studies indicated that the opposing roles of PRMTs in inducing or inhibiting the pathophysiology of CVDs mainly rely on their methylation modifications of various histones, transcription factors, and functional proteins. All of these studies suggest that PRMTs are crucial for maintaining cardiovascular homeostasis and may offer promising therapeutic possibilities for CVDs. However, their functions are context-dependent, varying according to cell type and pathological status.

## 5. Conclusions and Perspectives

This review summarizes the classification, structural features, and biological functions of methyltransferases, with a particular focus on PRMTs and their emerging roles in CVDs. We provide a comprehensive overview of the methyltransferase superfamily based on substrate specificity and structural features, highlighting universal roles of methylation in biological processes played by modulating proteins, DNA, RNA, and other biomolecules. By methylating histones and non-histone proteins to produce MMA, ADMA, or SDMA, the nine PRMTs exert broad control over transcription, RNA processing, signal transduction, and stress responses.

Currently, research on the functions of PRMTs in CVDs has made significant progress. The core mechanism is that PRMTs modulate downstream functional proteins by catalyzing arginine methylation of histones (such as H4R3, H3R8, H3R2), key transcription factors (such as NF-κB p65, ATF4, HOXA9, GATA4, PDCD4), and various modifying enzymes (such as p38 MAPK, CaMKII, p300, OGA). These modifications ultimately alter downstream effector proteins and affect lipid metabolism, inflammation, ER stress, cell proliferation and phenotypic switching, myocardial contraction, cytoskeleton-adhesion homeostasis, and ion channel function.

Among PRMTs, PRMT1 and PRMT5 are recognized as the most promising targets for CVD therapy, while PRMT4 is a clearly pathogenic target. However, PRMT functions are significantly context-dependent, varying across cell types and pathological states; therefore, non-specific manipulation may trigger bidirectional side effects. Accordingly, understanding such context-dependent regulatory patterns is essential for precise intervention.

Regarding targeting strategies, traditional pan-PRMT inhibitors and activators indiscriminately modulate multiple subtypes with highly conserved catalytic domains (especially the SAM-binding pocket), leading to serious off-target effects and toxicities. Therefore, there is an urgent demand to develop highly specific inhibitors and activators that accurately target particular subtypes. A more promising approach is to target the divergent N-terminal domains of each member, including the SH3 domain of PRMT2, the zinc-finger domain of PRMT3, the PH domain of PRMT4, the TIM-barrel domain of PRMT5, the N-terminal myristoylation domain of PRMT8, and the three TPR motifs of PRMT9. These regions can effectively circumvent the homology issues inherent to the conserved catalytic core. However, this approach faces challenges: N-terminal domains lack stable, deep small-molecule binding pockets for conventional small-molecule drug design, and systemic delivery cannot distinguish pathogenic from normal cells.

Given the context-dependent duality of PRMTs in CVDs, future therapies require both selective inhibitors (to block pathogenic actions) and precise activators (to preserve protective functions). Research should focus on the following directions: First, developing subtype-specific modulators that target unique N-terminal domains, despite druggability hurdles; second, integrating structural biology, computational design, nanodelivery, and cell-type-specific targeting to enhance precision and reduce toxicity. Ultimately, these efforts will pave the way for personalized therapies that block pathogenic pathways while retaining PRMT-mediated physiological protection.

In summary, as types of methyltransferase, PRMTs are important regulators of cardiovascular homeostasis and are expected to become highly promising targets for next-generation CVD therapies.

## Figures and Tables

**Table 1:** The molecular targets of PRMT1–PRMT7 in CVDs.

CVDs	PRMTs	Molecular Targets	References
IHD	PRMT1	ATF4, RAP1.	[129,130]
	PRMT2	ABCA1	[97,98]
	PRMT3	LXR	[100,102]
	PRMT4	VEGFR-2, caspase-3.	[123,127]
	PRMT5	SREBP1a, PDCD4, NF-κB p65, HOXA9, Kindlin-2, HIF-1α, ASK1, H3R8, H4R3, KLF4.	[104,105,109,112,119,121,122,125,126]
	PRMT7	p38 MAPK, HSP70.	[124,128]
HHD	PRMT1	CaMKII, SRSF1, cTnI.	[135,136,137]
	PRMT5	HOXA9, FILIP1L, GATA4, E2F-1, p300, H3R2, Smad3.	[140,141,142,143,146]
	PRMT6	H3R2	[149]
	PRMT7	β-Catenin	[150]
HCM	PRMT1	cTnI	[137]
DCM	PRMT1	EIF4A2	[155]
	PRMT5	OGA	[157]
ACM	PRMT1	desmoplakin	[158]
DIC	PRMT4	NRF2	[154]
	PRMT7	SOCS3	[161]
Arrhyth -mias	PRMT1	KCNQ1	[163]
	PRMT3	Nav1.5	[164]
	PRMT5	Nav1.5	[164]
AA	PRMT1	myocardin	[166]

## Data Availability

No new data were created or analyzed in this study. Data sharing is not applicable to this article.

## Abbreviations

The following abbreviations are used in this manuscript: 5-mTHF, 5-methyltetrahydrofolate; AA, aortic aneurysm; ABCA1, ATP-binding cassette transporter A1; ACM, arrhythmogenic cardiomyopathy; ADK, adenosine kinase; ADMA, asymmetric dimethylarginine; Ang II, angiotensin II; ASK1, apoptosis signal-regulating kinase 1; ATF4, activating transcription factor 4; ATP, adenosine triphosphate; B_12_, vitamin B_12_; BHMT, betaine homocysteine S-methyltransferase; BNP, natriuretic peptide; CaMKII, Ca^2+^/calmodulin-dependent kinase II; CARM1, coactivator-associated arginine methyltransferase 1; CIMT, carotid intima-media thickness; CMT, C-methyltransferase; COMT, catechol O-methyltransferase; CpG, cytosine-phosphate-guanine; cTnI, cardiac troponin I; CysG, siroheme synthase; DCM, dilated cardiomyopathy; DIC, doxorubicin-induced cardiomyopathy; DNMT, DNA methyltransferase; E2F-1, E2F transcription factor 1; EIF4A2, eukaryotic translation initiation factor 4A2; FILIP1L, filamin A interacting protein 1 like; GAR, glycine-arginine-rich; GATA4, GATA binding protein 4; H3R2, histone H3 arginine 2; H3R8, histone H3 arginine 8; H4R3, histone H4 arginine 3; HCM, hypertrophic cardiomyopathy; HHD, hypertensive heart disease; HIF-1α, hypoxia-inducible factor 1-α; HOXA9, Homeobox A9; HSP70, heat shock protein 70; IDR, intrinsically disordered region; IHD, ischemic heart disease; ISO, isoproterenol; KCNQ1, potassium voltage-gated channel subfamily q member 1; KLF4, Kruppel-like factor 4; lncRNA, long noncoding RNA; LXR, liver X receptor; m^1^A, 1-methyladenosine; m^3^C, 3-methylcytosine; m^5^C, 5-methylcytosine; m^6^A, 6-methyladenosine; m^7^G, 7-methylguanosine; MAT, methionine adenosyltransferase; MEP50, methylosome protein 50; miRNA, microRNA; MMA, monomethylarginine; mRNA, messenger RNA; MS, methionine synthase; mtcB, L-carnitine methyltransferase; MT, SAM-dependent methyltransferase; Nav1.5, sodium voltage-gated channel alpha subunit 5; NETs, neutrophil extracellular traps; NF-κB p65, nuclear factor NF-Kappa-B p65 subunit; NLRP3, NLR family pyrin domain containing 3; NMT, N-methyltransferase; NO, nitric oxide; NPMT, natural-product methyltransferase; Nrf2, nuclear factor erythroid 2-related factor 2; NSUN, NOL1/NOP2/SUN domain; OGA, N-acetyl-beta-glucosaminidase; OMT, O-methyltransferase; p38 MAPK, p38 mitogen activated protein kinase; PDCD4, programmed cell death 4; PE, phenylephrine; PGM, proline-glycine-methionine; PIP_2_, phosphatidylinositol 4,5-bisphosphate; PKMT, protein lysine methyltransferase; PNMT, phenylethanolamine N-methyltransferase; PPK, bnp; PRMT, protein arginine methyltransferase; RAP1, Ras-associated protein-1; ROS, reactive oxygen species; RPS2, ribosomal protein S2; rRNA, ribosomal RNA; SAM, S-adenosylmethionine; SAH, S-adenosylhomocysteine; SAHH, SAH hydrolase; SET, Su(var)3-9, enhancer of zeste, and trithorax; SF3B2, splicing factor 3b subunit 2; Sm, Smith antigen; SMN, survival motor neuron; SMT, S-methyltransferase; SN2, nucleophilic substitution; snRNA, small nuclear RNA; snoRNA, small nucleolar RNA; snRNP, small nuclear ribonucleoprotein; SOCS3, suppressor of cytokine signaling; SREBP1a, sterol regulatory element-binding protein 1a; SRSF1, serine/arginine-rich splicing factor 1; STAT1, signal transducer and activator of transcription 1; TAC, transverse aortic constriction; TGF-β, transforming growth factor-β; TDRD3, Tudor domain-containing 3; THF, tetrahydrofolate; tRNA, transfer RNA; TRMT, tRNA methyltransferase; VCAM-1, vascular cell adhesion molecule-1; YB1, Y-box binding protein 1; β-MyHC, β-myosin heavy chain.
